# Short-Term Treatment with Alirocumab, Flow-Dependent Dilatation of the Brachial Artery and Use of Magnetic Resonance Diffusion Tensor Imaging to Evaluate Vascular Structure: An Exploratory Pilot Study

**DOI:** 10.3390/biomedicines10010152

**Published:** 2022-01-11

**Authors:** Thomas Metzner, Deborah R. Leitner, Gudrun Dimsity, Felix Gunzer, Peter Opriessnig, Karin Mellitzer, Andrea Beck, Harald Sourij, Tatjana Stojakovic, Hannes Deutschmann, Winfried März, Ulf Landmesser, Marianne Brodmann, Gernot Reishofer, Hubert Scharnagl, Hermann Toplak, Günther Silbernagel

**Affiliations:** 1 Department of Internal Medicine, Division of Angiology, Medical University of Graz, 8036 Graz, Austria; metzner.thomas@gmx.at (T.M.); gudrun.dimsity@medunigraz.at (G.D.); marianne.brodmann@medunigraz.at (M.B.); guenther.silbernagel@medunigraz.at (G.S.); 2 Department of Medical Affairs, Sanofi-Aventis GmbH, 1100 Vienna, Austria; 3 Department of Internal Medicine, Division of Endocrinology and Diabetology, Medical University of Graz, 8036 Graz, Austria; deborah.leitner@gmx.at (D.R.L.); karin.mellitzer@yahoo.de (K.M.); Andrea.Beck@klinikum-graz.at (A.B.); ha.sourij@medunigraz.at (H.S.); 4 Department of Radiology, Clinical Division of Neuroradiology, Vascular and Interventional Radiology, Medical University of Graz, 8036 Graz, Austria; felix.gunzer@stud.medunigraz.at (F.G.); peter.opriessnig@alumni.tugraz.at (P.O.); hannes.deutschmann@medunigraz.at (H.D.); gernot.reishofer@medunigraz.at (G.R.); 5 Clinical Institute of Medical and Chemical Laboratory Diagnostics, University Hospital Graz, 8036 Graz, Austria; stojakovic@gmx.at; 6 Department of Internal Medicine 5 (Nephrology, Hypertensiology, Endocrinology, Diabetology, Rheumatology), Mannheim Medical Faculty, University of Heidelberg, 68167 Mannheim, Germany; winfried.maerz@synlab.com; 7 Synlab Academy, Synlab Holding Germany GmbH, 86156 Augsburg, Germany; 8 Clinical Institute of Medical and Chemical Laboratory Diagnostics, Medical University of Graz, 8036 Graz, Austria; hubert.scharnagl@medunigraz.at; 9 German Center for Cardiovascular Research (DZHK)-Partner Site Berlin, Department of Cardiology, Berlin Institute of Health, Charité University Medicine Berlin, 12200 Berlin, Germany; ulf.landmesser@charite.de

**Keywords:** alirocumab, PCSK9, lipids, magnetic resonance, endothelial function, ultrasound

## Abstract

Background: Short-term effects of alirocumab on vascular function have hardly been investigated. Moreover, there is a scarce of reliable non-invasive methods to evaluate atherosclerotic changes of the vasculature. The ALIROCKS trial was performed to address these issues using standard ultrasound-based procedures and a completely novel magnetic resonance-based imaging technique. Methods: A total of 24 patients with an indication for treatment with PCSK9 antibodies were recruited. There were 2 visits to the study site, the first before initiation of treatment with alirocumab and the second after 10 weeks of treatment. The key outcome measures included the change of carotid vessel wall fractional anisotropy, a novel magnetic resonance-based measure of vascular integrity, and the changes of carotid intima-media thickness and flow-dependent dilatation of the brachial artery measured with ultrasound. Results: A total of 19 patients completed the trial, 2 patients stopped treatment, 3 patients did not undergo the second visit due to the COVID pandemic. All of them had atherosclerotic vascular disease. Their mean (standard deviation) LDL-cholesterol concentration was 154 (85) mg/dL at baseline and was reduced by 76 (44) mg/dL in response to alirocumab treatment (*p* < 0.001, n = 19). P-selectin and vascular endothelial growth factors remained unchanged. Flow-dependent dilatation of the brachial artery (+41%, *p* = 0.241, n = 18), carotid intima-media thickness (*p* = 0.914, n = 18), and fractional anisotropy of the carotid artery (*p* = 0.358, n = 13) also did not significantly change. Conclusion: Despite a nominal amelioration for flow-dependent dilatation, significant effects of short-term treatment with alirocumab on vascular function were not detectable. More work would be needed to evaluate, whether fractional anisotropy may be useful in clinical atherosclerosis research.

## 1. Introduction

Elevated Low-density lipoprotein (LDL) cholesterol is associated with increased atherosclerotic plaque volume [1]. On the other hand, LDL-cholesterol lowering is instrumental to reduce atherosclerotic plaque volume [2,3]. Treatment with proprotein convertase subtilisin/kexin type 9 (PCSK9) antibodies reduces plasma LDL-cholesterol by as much as 60% [4,5,6]. In addition, treatment with PCSK9-antibodies has been demonstrated to be associated with plaque regression and higher plaque stability measured by intravascular ultrasound and optical coherence tomography [7,8,9,10]. However, intravascular ultrasound and optical coherence tomography both carry the risk of invasive procedures such as hematoma after an arterial puncture, infection, allergy against contrast media, or catheter-induced endothelial damage. Carotid intima-media thickness is commonly used for the non-invasive estimation of plaque formation [11,12]. This method is safe but imprecise. Brachial artery flow-dependent dilatation represents another ultrasound-based method that is considered a measure of the atherosclerotic process. However, it does not provide information on structural changes of the arteries [13,14]. Reliable non-invasive procedures to measure atherosclerotic changes are therefore needed [15].

We have introduced a completely novel method based on magnetic resonance diffusion tensor imaging to quantify structural changes of the carotid arteries. Ex-vivo, this method was demonstrated to have a high spatial resolution in visualizing collagen fiber bundles of the atherosclerotic fibrous cap [16]. In addition, this method appeared to provide information on vascular aging in a cohort of healthy males considering the inverse relationship between age and fractional anisotropy. This diffusion tensor imaging-based parameter is considered to reflect the proper alignment of collagen fiber bundles of the carotid arteries [17].

The present study was designed to explore the usability of this novel non-invasive magnetic resonance imaging method in patients with atherosclerotic vascular disease and to collect longitudinal clinical data on fractional anisotropy for the first time. Moreover, the objective was to investigate the effects of short-term treatment with alirocumab on brachial artery flow-dependent dilatation as well as inflammatory and vascular biomarkers.

## 2. Methods

### 2.1. Study Design

The ALIROCKS study (NCT03559309) is a prospective, monocentric, and longitudinal pilot study with 24 patients scheduled for treatment with alirocumab. Treatment with alirocumab was initiated based on the current guidelines for the treatment of dyslipidemia of the European Atherosclerosis Society and the European Society of Cardiology [18]. Inclusion criteria were atherosclerotic cardiovascular disease and elevated LDL-cholesterol despite maximum tolerated oral lipid-lowering therapy. Exclusion criteria were age <18 years, pregnancy, previous treatment with PCSK9-antibodies, and known contraindication for magnetic resonance imaging of the carotid artery (e.g., claustrophobia). There were 2 visits to the study site, the first before initiation of treatment with alirocumab and the second after 10 weeks of treatment. Diagnostic procedures of the first visit included a physical examination and a questionnaire on the medical history. Laboratory testing, ultrasound-based vascular imaging, and 2-dimensional cardiovascular magnetic resonance imaging of the carotid arteries were performed at the first and second visits.

The primary outcome measure was defined as the change of mean carotid vessel wall fractional anisotropy. Secondary outcome measures included the change of flow-dependent dilatation of the brachial artery, change of carotid intima-media thickness, and change of systemic inflammation in response to treatment with alirocumab. The clinical trial was conducted in accordance with the Declaration of Helsinki and was approved by the Austrian Federal Office for Safety in Health Care (EudraCT: 2018-000981-12) and the Ethics Committee of the Medical University of Graz (protocol number: 29-519 ex 16/17, approval date: 6 April 2018). All participants gave written informed consent.

### 2.2. Clinical Characterization

At baseline, demographics, and clinical characteristics such as age, sex, smoking habits, concomitant diseases, and lipid medication were documented. Familial hypercholesterolemia was reported according to medical history. Patients not receiving high-intensity statins defined as ≥40 mg atorvastatin or ≥20 mg rosuvastatin at baseline were categorized as having partial or complete statin intolerance.

### 2.3. Cardiovascular Magnetic Resonance Imaging

High-resolution diffusion tensor imaging was performed with a 2D diffusion acquisition scheme. A readout-segmented Echo-Planar-Imaging (rs-EPI MRI) sequence was used on a 3T whole-body MR scanner (Prisma fit, Siemens Medical Solutions, Erlangen, Germany) with a 2 × 4 channel multifunctional coil (NORAS MRI products GmbH, Höchberg, Germany) and FOV = 189 × 189 mm^2^, matrix = 346 × 346, slice thickness = 10 mm, TR = 2RR intervals, trigger delay = 50ms to acquire images in the diastolic phase, TE = 93 ms, GRAPPA = 2, number of readout segments = 9, b-values = 0, 200, 400, 600 s/mm^2^, acquisition time = ~12 min. 18 gradient directions were defined on a hemicycle within a plane oriented perpendicular to the longitudinal axis of the carotid artery. Fractional anisotropy is a scalar value without a unit between one and zero which describes the degree of anisotropy of a diffusion process (Figure S1). A value of one means that the process of diffusion is directed along one axis and is fully restricted along with all other directions, while a value of zero means that diffusion is isotropic or unrestricted in all directions. Lower values of fractional anisotropy are considered to reflect pathological changes of the carotid vessel wall structure [16,17]. The semiautomatic evaluation of fractional anisotropy was not conducted in a blinded design per study protocol. However, the person responsible for image analysis was not informed about the patient numbers and the time points of the measurements (baseline or week 10).

### 2.4. Laboratory Procedures

Blood samples were taken in the fasting state. LDL-cholesterol and HDL-cholesterol were assessed by lipoprotein-electrophoresis, cholesterol and triglycerides were measured by photometry. Apolipoproteins (Apo) and C-reactive protein (CRP) were measured with immunoturbidimetry, and interleukin-6 (IL-6) was measured with an electrochemiluminescence immunoassay. Monocyte chemoattractant protein-1 (MCP-1, by TECAN, Männedorf, Switzerland, ID: 30150435), Vascular endothelial growth factor (VEGF, by IBL, Minneapolis, MN, USA, ID: JP27171), and P-Selectin/CD62P (by Bio-Techne, Minneapolis, MN, USA, ID: DPSE00), were measured by enzyme-linked immunosorbent assays.

### 2.5. Intima-Media Thickness and Flow-Dependent Dilatation

All vascular ultrasound measurements were performed by a single experienced technician using a linear array transducer, 8–13 MHz (Sequoia 512, ACUSON Corp., Charleston Rd., Mountain View, CA, USA). Carotid intima-media thickness was defined as the mean of the left and right intima-media thickness which was each based on three different measurements in a one-centimeter-long segment of the common carotid artery. For the standardized measurement of flow-dependent dilatation of the brachial artery, a blood pressure cuff on the forearm was inflated for 5 min with a constant pressure of 50 mmHg above the systolic brachial artery blood pressure. The post-ischemic brachial arterial diameter was measured 45 s after cuff release. Flow-dependent dilatation was defined as the percent change of the brachial artery diameter in response to ischemia compared to the baseline diameter [13,14].

### 2.6. Statistical Analysis

The statistical design of this pilot trial was explorative and descriptive. The study duration and sample size were based on previous studies investigating the effects of short-term lipid-lowering on endothelial function [19,20,21,22,23,24]. Baseline characteristics are specified as numbers (percentages) in cases of categorical variables and as means (standard deviations) in cases of continuous variables. Changes in between baseline and week 10 were tested with the paired samples *t*-test and/or related samples Wilcoxon signed-rank test when Shapiro-Wilk analysis suggested non-normal distribution. The SPSS 26.0 statistical program was used (SPSS Inc., Chicago, IL, USA).

### 2.7. Monitoring

Source data verification was performed for carotid vessel wall fractional anisotropy assessed by cardiovascular magnetic resonance and for flow-dependent dilatation of the brachial artery measured by ultrasound. In addition, informed consent forms were monitored. The monitoring was conducted by an independent, external clinical research organization (MM Clinical Services & Consulting GmbH, Hart bei Graz, Austria).

## 3. Results

### 3.1. Study Population

The recruitment phase for the 24 participants lasted from 20 June 2018 to 30 January 2020. About 80% of the participants received 75 mg of alirocumab every two weeks and 20% received 150 mg alirocumab every two weeks. Two patients only injected alirocumab twice, at baseline and week 2. The reasons for their non-adherence were documented as drug access and communication barriers in the clinical routine. Four patients had minor changes in their oral lipid-lowering therapies during the trial. Among these, two patients stopped their red yeast rice product, one stopped ezetimibe, and another one reduced the oral combination therapy from 80 mg atorvastatin plus 10 mg ezetimibe to 20 mg atorvastatin monotherapy. Due to the COVID-19 pandemic and the subsequent implementation of national countermeasures restricting all hospital visits to essential interventions only, the clinical trial had to be terminated before the last 3 participants had completed the study. Hence, 19 patients completed the trial according to the protocol (Table 1). The mean (± standard deviation) treatment duration with alirocumab for participants who had completed the trial was 10.0 (±0.7) weeks.

### 3.2. Characteristics of Trial Participants

The cohort included middle-aged and elderly females and males. All 19 participants had documented coronary heart disease. Among these, 7 patients had poly-vascular disease with an additional diagnosis of cerebral and/or peripheral artery occlusive disease. The vast majority, 16 participants, had received the coronary intervention or bypass surgery. All 3 patients without prior coronary intervention or surgery had poly-vascular disease with coronary stenosis (50%) confirmed by cardiac computed tomography and carotid artery occlusive disease. One of the 3 patients without prior coronary intervention or surgery also had peripheral artery disease. Familial hypercholesterolemia was documented in 2 patients. A total of 16 study participants had partial or complete statin intolerance. Hence, very few participants received high-intensity statins at baseline. However, the proportion of patients taking ezetimibe as concomitant medication was high (Table 2 and Table S1).

### 3.3. Effects of Alirocumab on Lipids, Systemic Inflammation, and Vascular Biomarkers

Mean LDL-cholesterol was reduced by 76 mg/dL in response to treatment with alirocumab (relative reduction of 49%; Table 3 and Figure S2 of Supplementary Materials). Apo-B was reduced by 39%, cholesterol by 32%, and Lp(a) by 16%. Two patients had documented familial hypercholesterolemia and in one of them, baseline LDL-cholesterol was >400 mg/dL. Eight participants had Lp(a) values >50 mg/dL. In this subgroup Lp(a) was reduced from 94 mg/dL to 81 mg/dL resulting in a mean absolute reduction of 14 (12) mg/dL (*p* = 0.018). We did not detect changes in the systemic inflammatory biomarkers CRP and MCP-1 in response to treatment with alirocumab. There was only a minor but statistically significant reduction of IL-6. The vascular biomarkers P-selectin/CD62P and VEGF did not change in response to alirocumab treatment (Table 3).

### 3.4. Effects of Alirocumab on Vascular Structure and Function

**Figure 1:**
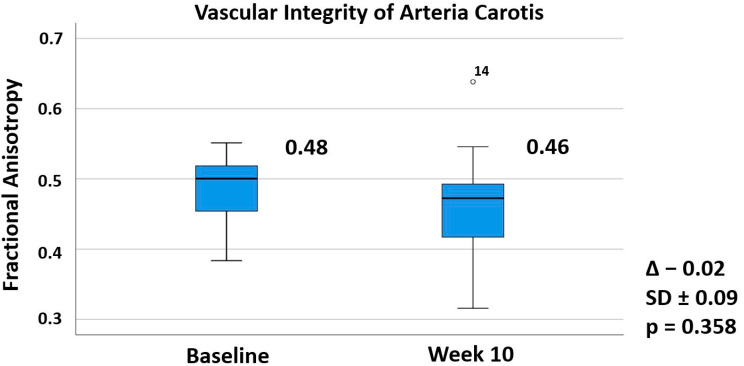
Effects of Alirocumab on a Novel Magnetic Resonance-based Vascular Parameter. Legend: Assessment of vascular integrity by evaluating fractional anisotropy of the carotid vessel wall. The numerical data shows mean values at baseline and week 10 of alirocumab treatment with mean change, standard deviation, and *p*-value, while boxplots graphically represent medians and interquartiles. The small circle with the number 14 at the right upper side of the figure identifies an outlier measurement of patient number 14 at week 10. Outliers are defined as values between 1.5× and 3× interquartile ranges from the end of a box. Fractional anisotropy is a scalar value without a unit between one and zero. Lower values of fractional anisotropy are considered to reflect pathological changes of the carotid vessel wall structure. Trial-completion analysis of patients with two valid assessments for statistical analysis (n = 13). Paired *t*-test with a two-sided *p*-value.

**Figure 2:**
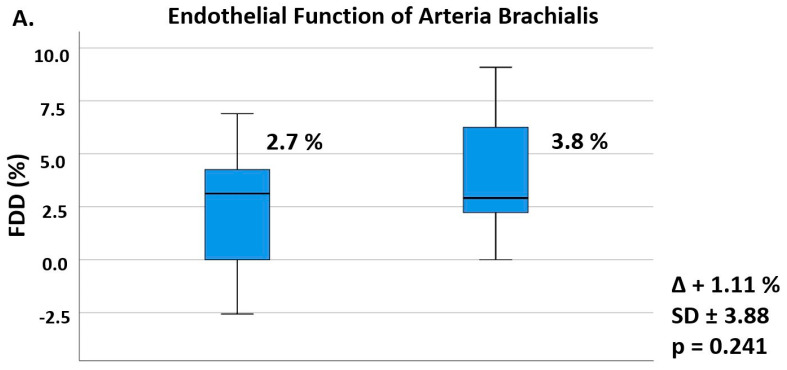

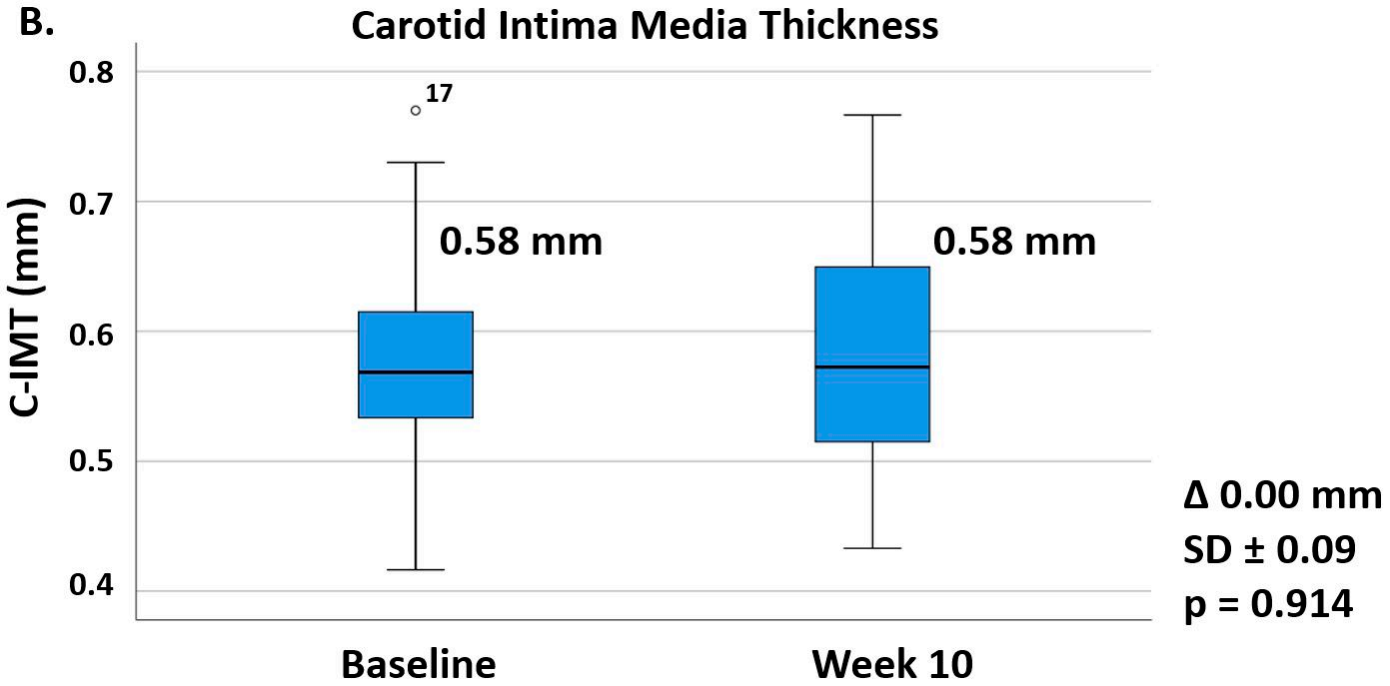
Effects of Alirocumab on Established Ultrasound-based Vascular Parameters. Legend: The numerical data shows mean values at baseline and after 10 weeks of alirocumab treatment with mean change, standard deviation, and *p*-value, while boxplots graphically represent medians and interquartiles. The small circle with the number 17 at the left upper side of the figure identifies an outlier measurement of patient number 17 at baseline. Outliers are defined as values between 1.5× and 3× interquartile ranges from the end of a box. Trial-completion analysis with two valid assessments for statistical analysis (n = 18); Paired *t*-test with a two-sided *p*-value. (**A**) Flow-dependent dilatation (FDD) of the arteria brachialis. (**B**) Carotid intima-media thickness (C-IMT) of the vessel wall.

The structural investigation of the carotid arteria using magnetic resonance and standard ultrasound-based imaging showed no changes in response to 10 weeks of treatment with alirocumab (Figure 1 and Figure 2). The endothelial function of the arteria brachialis also did not significantly change (+41%, *p* = 0.241). There was a trend towards an inverse relationship between LDL-cholesterol reduction and the change of flow-dependent dilatation (r = 0.402, *p* = 0.098; Figure S3 of Supplementary Materials). We obtained valid fractional anisotropy measurements for baseline and week 10 for 13 patients and valid flow-dependent dilatation and carotid intima-media thickness measurements for 18 patients. The considerable drop-out number for the magnetic resonance imaging resulted from one patient’s anatomy (adiposity with short neck), two patients with claustrophobia, and an outlier value that was excluded for analysis. The remaining two patients missed their scheduled visits. One measurement was missing for both ultrasound-based techniques.

## 4. Discussion

Fractional anisotropy is a completely novel magnetic resonance imaging-based parameter measuring the structural integrity of the vasculature. Due to anatomic issues (adiposity with short neck), claustrophobia, an outlier value, and the time-consuming measurement regimen, values were only obtained for a subgroup of the ALIROCKS study limiting the current clinical and scientific applicability of the method. During the ALIROCKS study, factional anisotropy did not significantly change, as expected. The short duration of treatment with alirocumab, which resulted in a 49% LDL-cholesterol reduction, and the low sample did not suggest significant changes in vascular structure. Moreover, there are no established cut-off values differentiating normal fractional anisotropy values from pathologic ones. The reproducibility of this magnetic resonance imaging method was analyzed in a previous study of our group in healthy subjects by providing the coefficients of variation. Four repeated measurements in four healthy male volunteers showed coefficients of variation between 2.5% and 5.4%. This previous cross-sectional evaluation in healthy subjects also demonstrated an inverse relationship between age and fractional anisotropy. These participants had fractional anisotropy ranging from around 0.70 at 27 years to 0.56 at 57 years of age [17]. The present population had a mean age of 66 years and a mean baseline fractional anisotropy of 0.48, which is consistent with the concept of a progressive decrease of fractional anisotropy with increasing age. Associations of fractional anisotropy with LDL cholesterol were not detectable in the present small cohort.

There is a scarce of non-invasive methods to reliably measure atherosclerosis. Using invasive methods such as optical coherence tomography, PCSK9-antibodies have been suggested to stabilize the fibrous cap of coronary atherosclerotic lesions and to reduce the size of lipid-rich plaques. Two single-center trials used optical coherence tomography to investigate the effects of PCSK9-antibodies vascular structure: Firstly, a retrospective study reported that treatment with evolocumab for 12 weeks increased fibrous-cap thickness and reduced the lipid-rich plaque proportion in 18 patients (intervention group) with acute coronary syndromes [9]. Secondly, a prospective randomized trial reported that treatment with alirocumab for 36 weeks also increased fibrous cap thickness and reduced the lipid content of plaques in 12 patients (intervention group) with coronary artery disease [10]. Most recently, the HUYGENS phase 3 trial investigated the effects of 52 weeks evolocumab treatment on fibrous cap thickness by optical coherence tomography. This double-blind study enrolled 161 patients with non-ST-elevation myocardial infarction who were randomized to statin treatment plus evolocumab versus statin treatment alone. Fibrous cap thickness significantly increased in the statin plus evolocumab group versus the statin treatment group [25]. The well-renowned GLAGOV trial demonstrated that 1 year of treatment with evolocumab reduces atheroma volume of the coronary arteries using intravascular ultrasound, another invasive method. This multi-center, randomized, and placebo-controlled study included 423 participants with coronary artery disease per group [7]. However, changes in coronary plaque composition were not detectable using virtual histology [26]. In agreement with the GLAGOV trial, the randomized, prospective ODYSSEY J-IVUS trial with 206 participants with acute coronary syndromes per group reported a nominal reduction of the total atheroma volume in response to 36 weeks of alirocumab treatment [8].

In addition to the completely novel magnetic resonance method, established ultrasound-based methods, namely carotid intima-media thickness and flow-mediated dilatation, were used in this study. Of relevance, intima-media thickness at baseline ranged from 0.42 to 0.77 mm (mean 0.58) and would not meet current recommendations for significant cardiovascular disease recently defined as ≥1.5 mm by the American Society of Echocardiography [11]. This observation also highlights the importance of sophisticated cardiovascular imaging in primary prevention patients with hypercholesterolemia [27]. Accordingly, the current guidelines for the treatment of dyslipidemia endorsed by the European Atherosclerosis Society and the European Society of Cardiology consider carotid intima-media thickness as inferior to the coronary artery calcium score or the detection of significant (>50%) stenoses (coronary angiography, computer tomography or carotid ultrasound) for very-high risk categorization [18]. Not surprisingly, carotid intima-media thickness also did not change in response to alirocumab due to the short treatment period, low sample size, and relatively low baseline intima-media thickness. Over a longer period of 12 months, a recent retrospective observational analysis suggested a reduction of carotid intima-media thickness by evolocumab in 229 patients with statin pre-treatment [28]. Moreover, carotid intima-media thickness was reduced by treatment with PCSK9-antibodies in addition to ongoing lipoprotein apheresis in only 14 heterozygous familial hypercholesterolemia patients. However, the participants of this study had higher mean baseline LDL-cholesterol of 197 mg/dL compared with the ALIROCKS study, markedly elevated lipoprotein(a) of 380 nmol/L, and a much longer treatment duration of 42 months versus 10 weeks in the ALIROCKS study.

The ALIROCKS trial showed a nominal amelioration (+41%) but no significant change of flow-dependent dilatation of the brachial artery in response to alirocumab. In contrast, 8 weeks of treatment with evolocumab increased flow-dependent dilatation in a small Italian cohort of 14 patients. Interestingly, the effect size was similar in the ALIROCKS trial but baseline values in the ALIROCKS study were lower compared with the Italian study. This difference in baseline flow-dependent dilatation values may be due to differences in patient characteristics and/or observer bias between the present study and the Italian study. All participants of the Italian study had a prior myocardial infarction and did not reach LDL-cholesterol goals despite a combination therapy of a high-dose statin and ezetimibe. Another recent study with 25 patients suffering from familial hypercholesterolemia (mean baseline LDL-cholesterol: 201 mg/dL, mean baseline Lp(a): 69 mg/dL) also showed amelioration of endothelial function in response to 12 weeks of treatment with PCSK9-antibodies. The reason for the difference in the results may be because all ALIROCKS participants had an advanced atherosclerotic cardiovascular disease but considerably lower baseline LDL cholesterol. Of note, the characteristics of the ALIROCKS participants were consistent with recent real-world evidence from Austria [6]. Finally, differences in the results compared with previous studies may also be due to methodological limitations of the flow-mediated dilatation method [29].

Another objective of the trial was to test the effects of treatment with alirocumab on inflammatory and vascular biomarkers. Whereas a pro-inflammatory role of PCSK9 in atherosclerosis has been suggested, clinical studies did not show a significant reduction of systemic inflammatory biomarkers by PCSK9-antibodies, even in myocardial infarction [30,31]. Consistent with previous analyses of interventional PCSK9-antibody trials we detected no changes for CRP and MCP-1 and only a modest reduction of IL-6 plasma levels [30]. In this context, it is of relevance to consider that the participants did not have elevated biomarkers of inflammation at inclusion into the study. On the other hand, arterial wall inflammation measured with positron emission tomography/computed tomography was attenuated by alirocumab without changes in circulating inflammatory markers [32]. While in-vitro investigations of the PCSK9-antibody evolocumab suggested a stimulation of angiogenesis by increased VEGF release, we could not detect changes of plasma VEGF by 10 weeks of alirocumab treatment in our clinical trial [33]. In agreement with an Italian study, we did also not detect changes of plasma P-selectin in response to short-term PCSK9-antibody treatment [34]. However, another measurement time point of this Italian trial after a longer treatment (12 months) showed a significant reduction of soluble plasma P-selectin, which may suggest a time-dependent treatment effect on this vascular biomarker [34].

It is a strength of the ALIROCKS study that a novel and innovative magnetic resonance-based method to visualize and evaluate the vascular structure of the carotid arteries was used. We also included high-quality ultrasound-based vascular imaging data acquired by a single well-trained technician. Moreover, the magnetic resonance and ultrasound data were monitored by an independent clinical research organization. Finally, we were able to provide “real world” data of patients with an indication for treatment with PCSK9-antibodies. We recruited patients with diverse vascular complications, concomitant disorders, lipid-lowering premedication, and lipid levels. This approach reduces the risk of selection bias but at the same time leads to a more heterogeneous collective with differences in medication as well as in locations and extent of atherosclerotic lesions. Such differences in baseline characteristics may also have an impact on the results of the ALIROCKS study.

It is a limitation of the ALIROCKS study that no control group is available. However, all participants had a medical indication for treatment with PCSK9-antibodies. Hence, it would have been unethical to withhold alirocumab from half of the participants. The magnetic resonance imaging method has not yet been implemented in patients with atherosclerosis and comparisons with healthy individuals have not been made. Therefore, reference values for fractional anisotropy values in atherosclerotic vessels versus those vessels without atherosclerosis are currently not available. The relatively low sample size may also be regarded as a weak point of the ALIROCKS study. Nevertheless, beneficial effects of short-term lipid-lowering therapy on vascular function have previously been documented in studies with similar or smaller sample sizes and with even shorter treatment duration [19,20,21,22,23,24]. On the other hand, the duration of the intervention was too short to expect significant effects on fractional anisotropy of the carotid artery and carotid intima-media thickness. However, the aim was to gain and present clinical experience and longitudinal data with the novel magnetic resonance imaging method.

To conclude, endothelial function measured with flow-dependent dilatation of the brachial artery did not significantly change in response to short-term treatment with alirocumab. More work would be needed to evaluate whether fractional anisotropy may be useful in clinical atherosclerosis research.

## Figures and Tables

**Table 1:** Study Population and Alirocumab Dosage.

Definition	Population (N)	Alirocumab 75 mg (%)	Alirocumab 150 mg (%)
Recruitment	24	19 (79.2)	5 (20.8)
Completion	19	16 (84.2)	3 (15.8)

Legend: Trial completion population (n = 19) includes patients attending their baseline plus week 10 visit and injected alirocumab as planned. Three patients could not conduct their scheduled week 10 visit due to COVID-19 countermeasures. Two patients injected alirocumab only twice at baseline and at week 2 but not beyond.

**Table 2:** Characteristics of Trial Participants (Trial Completion).

Characteristic	Alirocumab (N = 19)
Age-yr	66 (9)
Female sex-n (%)	9 (47.4)
Male sex-n (%)	10 (52.6)
Smoker ^a^-n (%)	9 (47.4)
Current Smoker ^b^-n (%)	4 (21.1)
Concomitant Diseases-n (%)	
Cardiovascular Disease	19 (100)
Coronary Heart Disease	19 (100)
Coronary Intervention or Surgery	16 (84.2)
Documentation of Coronary Stenosis ^c^	3 (15.8)
Peripheral Artery Disease	3 (15.8)
Cerebral Artery Disease	6 (31.6)
Chronic Kidney Disease	4 (21.1)
Familial Hypercholesterolaemia ^d^	2 (10.5)
Adiposity	4 (21.1)
Type-2 Diabetes Mellitus	4 (21.1)
Type-1 Diabetes Mellitus	0 (0)
Hypertension	15 (78.9)
Number of prior Cardiovascular Events ^e^-n (%)	
Three	2 (10.5)
Two	5 (26.3)
One	9 (47.4)
Zero	3 (15.8)
Concomitant Lipid Medication–n (%)	
High-Intensity Statins ^f^	3 (15.8)
Statins	5 (26.3)
Ezetimibe	13 (68.4)
Dietary Supplements ^g^	5 (26.3)
Statin Intolerance ^h^	16 (84.2)

Legend: Values are numbers (percentages) or means (standard deviations) for categorical and continuous variables, respectively. ^a^ Current or former smoker. ^b^ Documented as a current smoker, or no stop date documented. ^c^ Confirmed by cardiac computed tomography but without documentation of prior cardiovascular event (e.g., stroke, myocardial infarction, or percutaneous intervention). ^d^ According to medical records. ^e^ Documented as a stent, balloon, coronary artery bypass graft, myocardial infarction, or percutaneous intervention, prior strokes/transient ischemic attacks. ^f^ Documented as ≥40 mg of atorvastatin or ≥20 mg of rosuvastatin. ^g^ Exclusively red yeast rice combination products (monacolin K). ^h^ Patients that did not receive high-intensity statins at baseline (includes partial or complete intolerance).

**Table 3:** Effects of Alirocumab on Lipids, Systemic Inflammation, and Vascular Biomarkers.

Parameters	Baseline	±SD	Week 10	±SD	Absolute Change	±SD	Relative Change (%)	*p*-Value
Standard Lipids ^a^								
LDL-Cholesterol (mg/dL)	154	85	78	91	−76	44	−49	<0.001
Apo-B (mg/dL)	124	56	76	65	−48	28	−39	<0.001
Cholesterol (mg/dL)	244	104	166	102	−78	41	−32	<0.001
Lp(a) (mg/dL)	51	43	43	39	−8	11	−16	0.007
Triglycerides (mg/dL)	148	87	132	65	−17	73	−11	0.332
HDL-Cholesterol (mg/dL)	50	13	59	16	+9	15	+18	0.018
Systemic Inflammation ^b^								
CRP (mg/L)	2.7	3.5	2.6	3.1	−0.1	1.7	−4	0.902
IL-6 (pg/mL)	3.1	1.7	2.7	1.4	−0.5	0.9	−13	0.030
MCP-1 (pg/mL)	617	219	623	188	+6.2	99	+1	0.789
Vascular Biomarkers ^c^								
VEGF (pg/dL)	6.2	6.1	7.5	7.8	+1.3	5.7	+21	0.409
P-Selectin/CD62P (ng/mL)	35.4	8.0	35.5	10.2	+0.1	7.4	+0.3	0.963

Legend: Table shows mean values and changes; Trial-completion analysis (n = 19); Paired *t*-test with a two-sided *p*-value. ^a^ LDL-Cholesterol and HDL-Cholesterol were assessed by lipid-electrophoresis; triglycerides and total cholesterol were measured by photometry; lipoproteins were determined by immunoturbidimetry. Units are mg/dL. ^b^ CRP was measured with immunoturbidimetry; IL-6 was measured with an electrochemiluminescence immunoassay; MCP-1 was measured by enzyme-linked immunosorbent assay. ^c^ Parameters were measured by enzyme-linked immunosorbent assays. VEGF: Five patients had values below the limit of detection (n = 14).

## Data Availability

The data presented in this study are available on specific request from the corresponding author.

## Supplementary Materials

The following are available online at https://www.mdpi.com/article/10.3390/biomedicines10010152/s1. Figure S1. Illustration of Fractional Anisotropy Evaluation by Magnetic Resonance Imaging; Figure S2. Plasma LDL-cholesterol Levels at Baseline and after 10 weeks of Alirocumab Treatment; Figure S3. Correlation of Alirocumab Treatment Effect on Flow-dependent Dilatation of the Arte-ria Brachialis and LDL-Cholesterol Lowering; Table S1. Characteristics of Trial Participants according to defined Study Populations.

## Abbreviations and Acronyms

Apo	Apolipoprotein
C-IMT	Carotid-Intima-media Thickness
COVID-19	Coronavirus Disease-2019
CRP	C-Reactive Protein
FDD	Flow-Dependent Dilatation
HDL	High-Density Lipoprotein
IL-6	Interleukin-6
Lp(a)	Lipoprotein-a particles
LDL	Low-Density Lipoprotein
MCP-1	Monocyte Chemoattractant Protein-1
MRI	Magnetic Resonance Imaging
PCSK9	Proprotein Convertase Subtilisin/Kexin type 9
VEGF	Vascular Endothelial Growth Factor
