# Supplementary material for: Short-Term Treatment with Alirocumab, Flow-Dependent Dilatation of the Brachial Artery and Use of Magnetic Resonance Diffusion Tensor Imaging to Evaluate Vascular Structure: An Exploratory Pilot Study
Source: Biomedicines. 2022 Jan 11;10(1):152. doi: 10.3390/biomedicines10010152 (PMC8773704; doi:10.3390/biomedicines10010152)
Supplement: Supplementary file 1 [file biomedicines-10-00152-s001.zip › biomedicines-1511250-sm.pdf]

Supplementary Materials

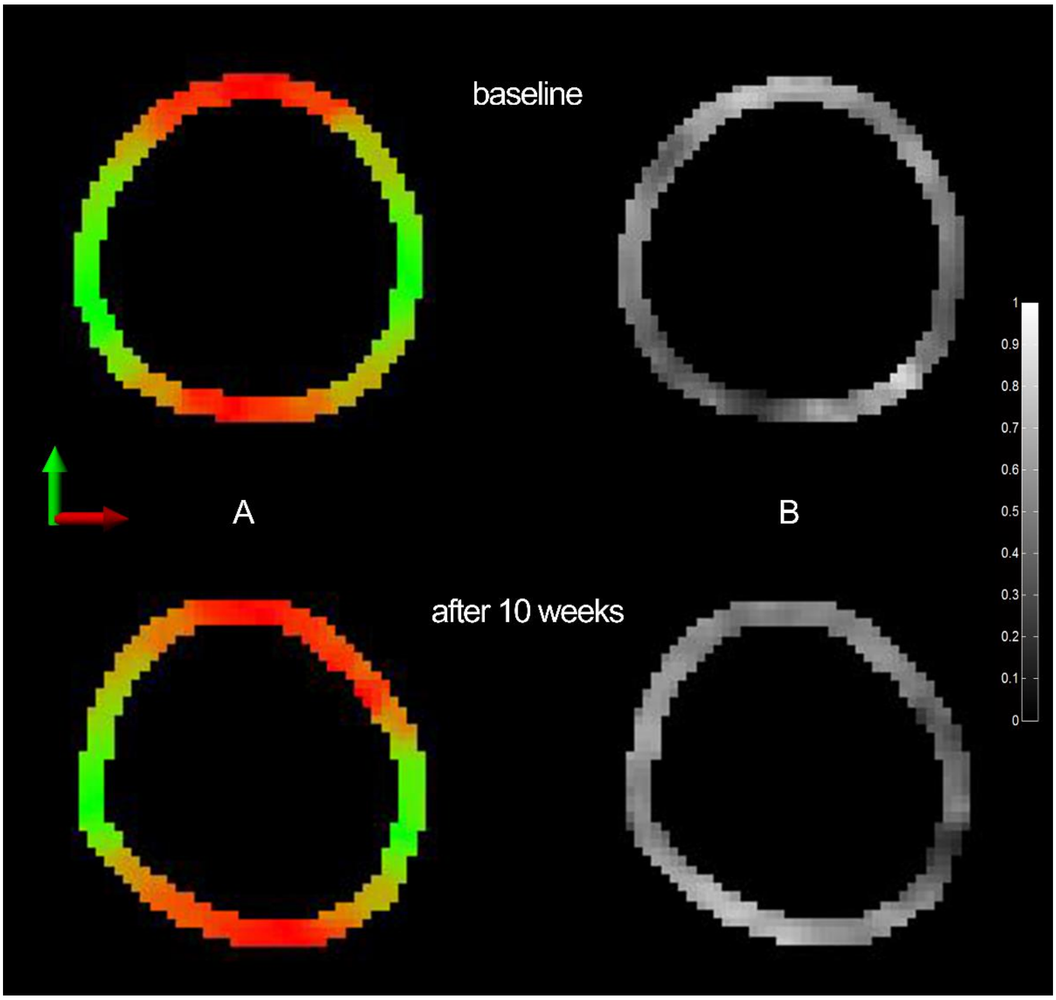

Figure S1. Illustration of Fractional Anisotropy Evaluation by Magnetic Resonance Imaging. Legend: The left column (**A**) shows colour coded vector images of the principal diffusion tensor direction with the corresponding grey-scale fractional anisotropy maps in the right column (**B**). The upper row shows the baseline measurement prior to the administration of alirocumab for a single participant (mean FA = 0.500). The lower row shows the imaging result for a single participant after 10 weeks of intervention (mean FA = 0.516). Fractional anisotropy (FA) is a scalar value without unit between one and zero (right grey-scale). A value of one means that the process of diffusion is directed along one axis and is fully restricted along all other directions, while a value of zero means that diffusion is isotropic or unrestricted in all directions. Lower values of fractional anisotropy are considered to reflect pathological changes of the carotid vessel wall structure. In this example of a single patient for illustration purposes, the fractional anisotropy values did not change in response to short-term alirocumab treatment meaning that no structural changes of carotid vessel wall could be detected.

Table S1. Characteristics of Trial Participants according to defined Study Populations.

Characteristic	All Recruited (N=24)	Trial Completion (N=19)
Age - yr	66 (9)	66 (9)
Female sex - n (%)	9 (37.5)	9 (47.4)
Male sex - n (%)	15 (62.5)	10 (52.6)
Smoker [a] - n (%)	12 (50)	9 (47.4)
Current Smoker [b] - n (%)	5 (20.8)	4 (21.1)
Concomitant Diseases - n (%)		
Cardiovascular Disease	24 (100)	19 (100)
a. Coronary Heart Disease	23 (95.8)	19 (100)
Coronary Intervention or Surgery	19 (79.2)	16 (84.2)
Documentation of Coronary Stenosis [c]	4 (16.7)	3 (15.8)
b. Peripheral Artery Disease	3 (12.5)	3 (15.8)
c. Cerebral Artery Disease	8 (33.3)	6 (31.6)
Chronic Kidney Disease	5 (20.8)	4 (21.1)
Familial Hypercholesterolaemia [d]	4 (16.7)	2 (10.5)
Adiposity	4 (16.7)	4 (21.1)
Type-2 Diabetes Mellitus	4 (16.7)	4 (21.1)
Type-1 Diabetes Mellitus	0 (0)	0 (0)
Hypertension	19 (79.2)	15 (78.9)
Number of prior Cardiovascular Events [e] - n (%)		
Three	2 (8.3)	2 (10.5)
Two	6 (25)	5 (26.3)
One	12 (50)	9 (47.4)
Zero	4 (16.7)	3 (15.8)
Concomitant Lipid Medication – n (%)		
High-Intensity Statins [f]	5 (20.8)	3 (15.8)
Statins	7 (29.2)	5 (26.3)
Ezetimibe	15 (62.5)	13 (68.4)
Dietary Supplements [g]	6 (25)	5 (26.3)
Statin Intolerance [h]	19 (79.2)	16 (84.2)

Legend: Values are numbers (percentages) or means (standard deviations) for categorical and continuous variables, respectively. [a] Current or former smoker. [b] Documented as current smoker or no stop date documented. [c] Confirmed by cardiac computed tomography but without documentation of prior cardiovascular event (e.g., stroke, myocardial infarction, or percutaneous intervention). [d] According to medical records. [e] Documented as stent, balloon, coronary artery bypass graft, myocardial infarction, or percutaneous intervention, prior strokes/transient ischemic attacks. [f] Documented as ≥ 40 mg of atorvastatin or ≥ 20 mg of rosuvastatin. [g] Exclusively red yeast rice combination products (monacolin K). [h] Patients that did not receive high-intensity statins at baseline (includes partial or complete intolerance).

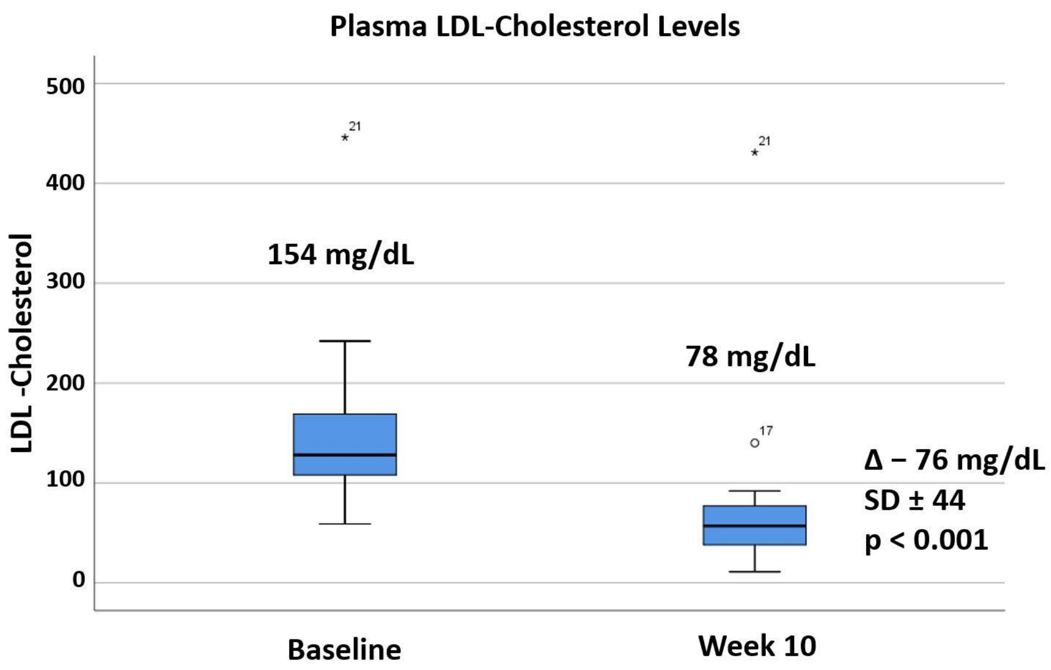

Figure S2. Plasma LDL-cholesterol Levels at Baseline and after 10 weeks of Alirocumab Treatment. Legend: Figure shows trial-completion analysis (N = 19); Paired t-test with two-sided *p*-value; SD = Standard deviation.

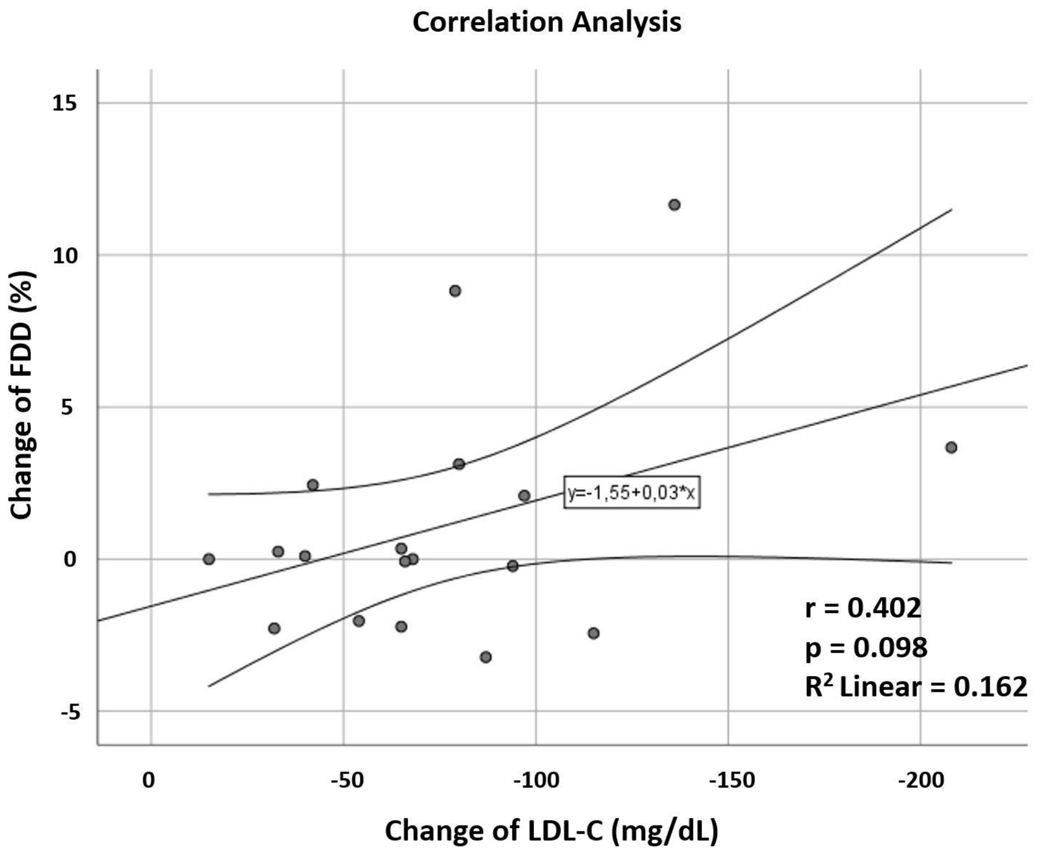

Figure S3. Correlation of Alirocumab Treatment Effect on Flow-dependent Dilatation of the Arteria Brachialis and LDL-Cholesterol Lowering. Legend: Figure shows trial-completion analysis (N = 19), one patient missed his scheduled ultrasound imaging visit. Pearson bivariate correlation analysis (r). FDD: Flow-dependent dilatation. .
